# Supplementary material for: Dual Switch in Lipid Metabolism in Cervical Epithelial Cells during Dysplasia Development Observed Using Raman Microscopy and Molecular Methods
Source: Cancers (Basel). 2021 Apr 21;13(9):1997. doi: 10.3390/cancers13091997 (PMC8122332; doi:10.3390/cancers13091997)
Supplement: Supplementary file 1 [file cancers-13-01997-s001.zip › cancers-1179496-supplementary.pdf]

Supplementary materials

Dual Switch in Lipid Metabolism in Cervical Epithelial Cells During Dysplasia Development Observed Using Raman Microscopy and Molecular Methods

Katarzyna Sitarz[1,2], Krzysztof Czamara[3], Joanna Bialecka[4], Malgorzata Klimek[5], Slawa Szostek[1*] and Agnieszka Kaczor[2,3*]

1 Jagiellonian University Medical College, Faculty of Medicine, Chair of Microbiology, Department of Molecular Medical Microbiology, 18 Czysta Street, 31-121 Krakow, Poland; katarzyna.sitarz@doctoral.uj.edu.pl (K.S.).
2 Jagiellonian University, Faculty of Chemistry, 2 Gronostajowa Street, 30-387 Krakow, Poland.
3 Jagiellonian University, Jagiellonian Centre for Experimental Therapeutics (JCET), 14 Bobrzynskiego Street, 30-348 Krakow, Poland; krzysztof.czamara@uj.edu,pl (K.C.).
4 Centre of Microbiological Research and Autovaccines, 17 Slawkowska Street, 31-016 Krakow, Poland; joanna.bialecka@gmail.com (J.B.).
5 Clinic of Radiotherapy, Maria Sklodowska-Curie Institute – Oncology Center, 11 Garncarska Street, 31-115 Krakow, Poland; malgorzata.klimek@onkologia.krakow.pl (M.K.).
* Correspondence: Agnieszka Kaczor (agnieszka.kaczor@uj.edu.pl) and Slawa Szostek (slawa.szostek@uj.edu.pl)

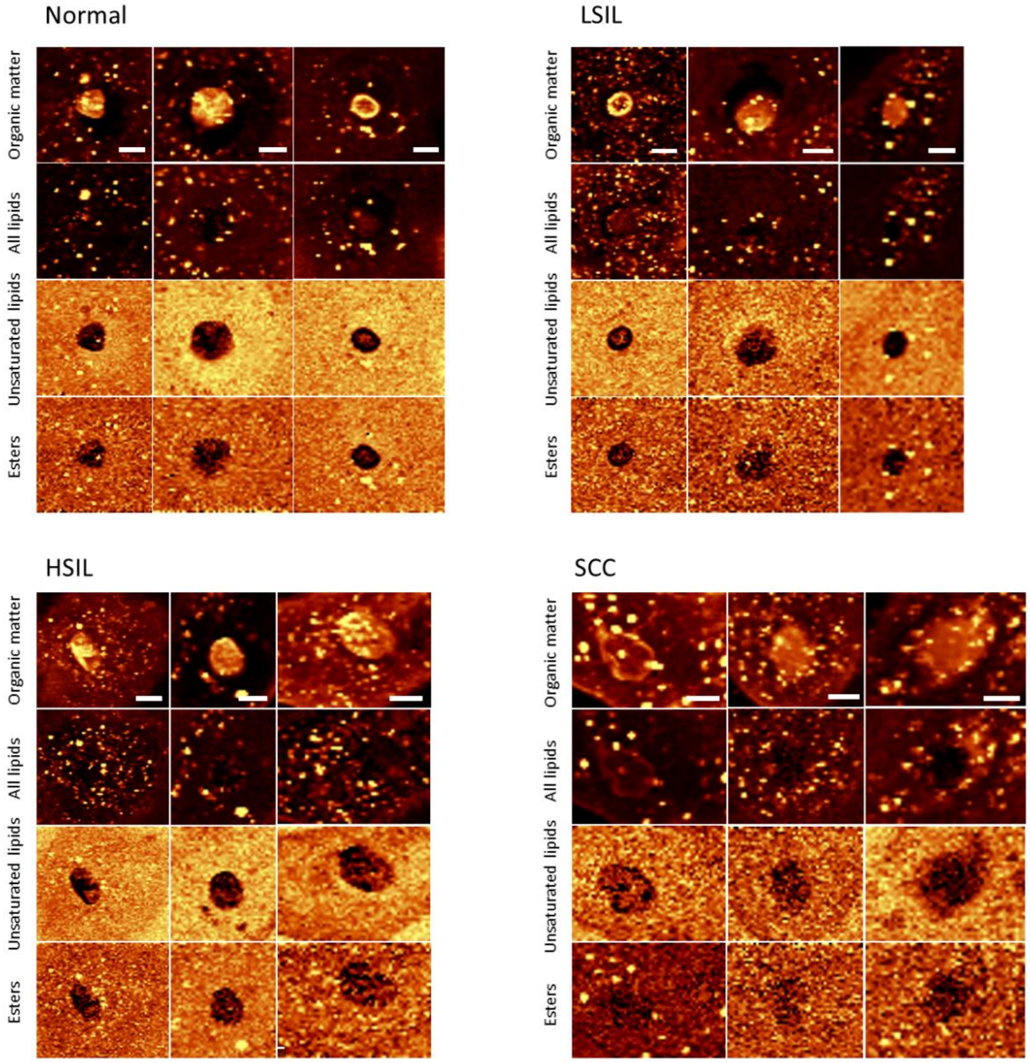

Figure S1. Subcellular distribution of lipids in cervical epithelial cells divided into following groups: normal, LSIL, HSIL and SCC. Representative Raman images of cervical epithelial cells obtained by integration in the spectral regions: 2830–3030 cm^{-1} (all organic matter), 2830–2900 cm^{-1} (total lipids), 2990-3020 cm^{-1} (unsaturated lipids) and 1715-1760 cm^{-1} (esters).

Table S1. The number of patients in studied groups.

Analyzed group	HPVhr status	Number of patients
N	HPVhr-	22
	HPVhr+	6
LSIL	HPVhr-	6
	HPVhr+	6
HSIL	HPVhr-	6
	HPVhr+	6
SCC[a]	HPVhr-	0
	HPVhr+	11

[a]histopathologically confirmed squamous cell carcinoma

Table S2. Percentage of HPV-16 and HPV-18 positive samples among HPVhr positive samples in individual groups.

Analyzed group	Percentage of positive samples
N	HPV-16: 17%
	HPV-18: 0%
LSIL	HPV-16: 33%
	HPV-18: 17%
HSIL	HPV-16: 67%
	HPV-18: 33%
SCC	HPV-16: 100%
	HPV-18: 0%

Table S3. Raman bands from publication and their assignments.

Raman band (cm^{-1})	Assignment
481	Glycogen skeletal modes,β(CCC)[1]
786	Ring breathing modes in the DNA&RNA bases - T,C[2]
858	Glycogen ν(COC), β(CC), ring breathing, β(CH) of C-1[1]
937	Glycogen ν_s(COC) of α-D-(1→6) glycosidic linkages, ring def. [1]
1266	Lipids δ(=CH)[3]
1304	Lipids τ(CH_2)[3]
1340	Nucleic acids ν(C-N) in the DNA&RNA bases - A,G[2]
1450	Lipids β(CH_2/CH_3)[3]
1664	Lipids ν(C=C)[3]
1747	Lipids ν(C=O)[3]
2851	Lipids ν_s(CH_2)[3]
2924	Lipids ν_s(CH_3)[3]
3006	Lipids ν(=CH)[3]

[1]Wiercigroch, E; Szafraniec, E; Czamara, K; Pacia, MZ; Majzner, K; Kochan, K; Kaczor, A; Baranska, M; Malek, K. *Raman and infrared spectroscopy of carbohydrates: A review*. Spectrochimica Acta Part A: Molecular and Biomolecular Spectroscopy 2017, 185, 317–335.

[2]Sofińska, K.; Wilkosz, N.; Szymoński, M.; Lipiec, E. *Molecular Spectroscopic Markers of DNA Damage.* Molecules 2020, 25, 561.

[3]Czamara, K.; Majzner, K.; Pacia, M.Z.; Kochan, K.; Kaczor, A.; Baranska, M. *Raman Spectroscopy of Lipids: A Review*. J. Raman Spectrosc. 2015, 46, 4–20.

Table S4. Lipid content in studied epithelial cervical cells. Lipid content calculated based on the relative intensity of the Raman band in the range of 2830-3000 cm^{-1}.

Classification	Number of cells	Relative lipid content	Statistical significance
**N/HPV**hr-	71	Mean: 34.92<br>SD: 1.46<br>SE: 0.17	LSIL/HPVhr- ***<br>LSIL/HPVhr+ ***<br>HSIL/HPVhr- ***<br>HSIL/HPVhr+ **<br>SCC/HPVhr+ ***
**N/HPV**hr**+**	15	Mean: 34.34<br>SD: 1.34<br>SE: 0.35	LSIL/HPVhr- **<br>LSIL/HPVhr+ ***<br>HSIL/HPVhr- *<br>HSIL/HPVhr+ ***<br>SCC/HPVhr+ ***
**LSIL/HPV**hr-	30	Mean: 31.23<br>SD: 3.29<br>SE: 0.60	N/HPVhr-***<br>N/HPVhr+**<br>HSIL/HPVhr+***<br>SCC/HPVhr+***
**LSIL/HPV**hr**+**	19	Mean: 29.93<br>SD: 3.15<br>SE: 0.72	N/HPVhr-***<br>N/HPVhr+***<br>HSIL/HPVhr-**<br>HSIL/HPVhr+***<br>SCC/HPVhr+***
**HSIL/HPV**hr-	21	Mean: 32.82<br>SD: 2.06<br>SE: 0.45	N/HPVhr-***<br>N/HPVhr+*<br>LSIL/HPVhr+**<br>HSIL/HPVhr+***<br>SCC/HPVhr+***
**HSIL/HPV**hr**+**	20	Mean: 36.07<br>SD: 1.27<br>SE: 0.28	N/HPVhr-**<br>N/HPVhr+***<br>LSIL/HPVhr-***<br>LSIL/HPVhr+***<br>HSIL/HPVhr-***<br>SCC/HPVhr+***
**SCC/HPV**hr**+**	21	Mean: 36.87<br>SD: 0.83<br>SE: 0.18	N/HPVhr-***<br>N/HPVhr+***<br>LSIL/HPVhr-***<br>LSIL/HPVhr+***<br>HSIL/HPVhr-***<br>HSIL/HPVhr+***

Table S5. Lipid unsaturation in lipid droplets of studied epithelial cervical cells. Lipid unsaturation calculated based on the Raman integral intensity ratio of the bands at 1270/1300 cm^{-1}.

Classification	Number of cells	Lipid unsaturation	Statistical significance
**N**	35	Mean: 0.41<br>SD: 0.09<br>SE: 0.01	LSIL**<br>SCC*
**LSIL**	15	Mean: 0.49<br>SD: 0.07<br>SE: 0.02	N**<br>HSIL*<br>SCC***
**HSIL**	21	Mean: 0.44<br>SD: 0.11<br>SE: 0.02	LSIL*<br>SCC*
**SCC**	11	Mean: 0.36<br>SD: 0.06<br>SE: 0.02	N*<br>LSIL***<br>HSIL*

Table S6. CpG island methylation data of studied *SREBF1* gene fragment.

Classification	Number of samples	Number of methylated samples	Number of CpG methylations in methylated samples (samples x methylations)	Level of methylation	Statistical significance	Level of methylation only in methylated samples
**N**	28	12	8 x 1; 3 x 2; 1 x 3	Mean: 0.61<br>SD: 0.83<br>SE: 0.16	-	Mean: 1.42<br>SD: 0.67<br>SE: 0.19
**LSIL**	12	8	2 x 1; 4 x 2; 2 x 3	Mean: 1.33<br>SD: 1.15<br>SE: 0.33	SCC*	Mean: 2.00<br>SD: 0.76<br>SE: 0.27
**HSIL**	12	4	2 x 1; 2 x 2	Mean: 0.50<br>SD: 0.80<br>SE: 0.23	-	Mean: 1.50<br>SD: 0.58<br>SE: 0.29
**SCC**	11	2	1 x 1; 1 x 5	Mean: 0.55<br>SD: 0.51<br>SE: 0.45	LSIL*	Mean: 3.00<br>SD: 2.83<br>SE: 2.00

Table S7. Level of mtDNA copies.

Classification	Number of measurements	Relative mtDNA level	Statistical significance
**N/HPV**hr-	43	Mean: 1.21<br>SD: 0.77<br>SE: 0.12	LSIL/HPVhr-**<br>HSIL/HPVhr+ ***<br>SCC/HPVhr+ ***
**N/HPV**hr**+**	12	Mean: 1.76<br>SD: 0.67<br>SE: 0.48	-
**LSIL/HPV**hr-	11	Mean: 1.40<br>SD: 1.22<br>SE: 0.37	N/HPVhr-**<br>HSIL/HPVhr+*
**LSIL/HPV**hr**+**	12	Mean: 1.16<br>SD: 0.80<br>SE: 0.23	HSIL/HPVhr+**<br>SCC/HPVhr+**
**HSIL/HPV**hr-	12	Mean: 2.09<br>SD: 1.59<br>SE: 0.46	-
**HSIL/HPV**hr**+**	12	Mean: 2.76<br>SD: 1.39<br>SE: 0.40	N/HPVhr-***<br>LSIL/HPVhr-*<br>LSIL/HPVhr+**
**SCC/HPV**hr**+**	21	Mean: 2.24<br>SD: 1.53<br>SE: 0.33	N/HPVhr-***<br>LSIL/HPVhr+**
